# Supplementary material for: Patterns of Intron Gain and Loss in Fungi
Source: PLoS Biol. 2004 Nov 30;2(12):e422. doi: 10.1371/journal.pbio.0020422 (PMC532390; doi:10.1371/journal.pbio.0020422)
Supplement: Table S1 — Also available at http://genes.mit.edu/NielsenEtAl/. (4.3 MB ZIP). [file pbio.0020422.st001.zip › NielsenEtAl/html/9.html]

AN2982.1.NCU09321.1.MG04371.1.FG01043.1


```
 CLUSTAL W (1.82) Multiple Sequence Alignments - Introns Inserted


Sequence 1: NCU09321.1	595 aa
Sequence 2: MG04371.1	608 aa
Sequence 3: FG01043.1	724 aa
Sequence 4: AN2982.1	516 aa
Alignment Length: 810 aa
Number Identitical Residues: 162 aa
Alignment Score (without introns) 9761


MG04371.1 	------------------------------------------------------------
NCU09321.1	------------------------------------------------------------
FG01043.1 	MPATSSLEEQAIDREYPQQQRQHHRRHQELEPEPRQFSFRSPNRNEAVPQSQLDNQQDTG
AN2982.1  	------------------------------------------------------------
          	                                                            

MG04371.1 	------------------------------------------------------------
NCU09321.1	------------------------------------------------------------
FG01043.1 	SLSYSQQHYRPQNSSLSHTQSNSRLVVPSVSGVRSTSPAPSVSPLPTPSISPSASVVSID
AN2982.1  	------------------------------------------------------------
          	                                                            

MG04371.1 	--------------------------------------------------------MATW
NCU09321.1	--MTDSIPPTDGKTPDNTCAPLEPRQ-----------------KYSTTQSPFPDFEFPVE
FG01043.1 	ASYSSRDPSSPIQTPSSTSAEAHDSHPQRDSSEHNSSERGSTDSYSYLRRERNSSNMATW
AN2982.1  	-----------------------------------------------------MPQAARW
          	                                                       . .  

MG04371.1 	SGRAAIKGGSDTMRMV------LLTCNAVGVT~FTWGVEMTY1CTPYLLSLGLTKGQTSL
NCU09321.1	QGNPSYQGEKSSDRHCKEEEEGLSPNQWVASG~EQEDNSGSY~SEAENVEMSTWRGSPSV
FG01043.1 	SGQPAIRGNSEAVRMI------LLCFVTIGIT2FTWGIEMTY1CTPYLLNLGLTKSNTSL
AN2982.1  	VGSPSIKGRTESMRMA------LLTFSLLGLQ2FTWGIEMTC1-----------------
          	 * .: :* ..: *        *     :.      . . :                   

MG04371.1 	VWIAGPLSGLIVQPVIG---~---------~VISDTWTSKWGRRRPFIMMCSVIVAMGLL
NCU09321.1	KGSTETMRMLLLTCVSVGIT2FTWGVEMTC1VVADQSKSRWGRRRPVIVIGSIITALALM
FG01043.1 	VWIAGPLSGLVVQPVVG---~---------~VIADESKSKWGRRRPLMVVGSIIVAISLL
AN2982.1  	--------------------~---------~VIADRSRSKWGRRRPFMIGGSLIVTVCLL
          	                               *::*   *:******.::  *:*.:: *:

MG04371.1 	TLGFTRDIVSFFIPSSAAGAAAAAAAEVPTA~IRRDHPENNGFTGFLTIALAVLALYTTD
NCU09321.1	ALGFTKEIVAYFIWD-------------PTY~AR-----------ACTIAVAVLSLYCVD
FG01043.1 	ILGFTREIIGYFITD-------------EEA~AKR-----------PTIVLAVLAIYVVD
AN2982.1  	VLGWTTEIVGVFVKD-------------AEK0AAS-----------VTIALAVLSIYAVD
          	 **:* :*:. *: .                                **.:***::* .*

MG04371.1 	FAINA-----V~MSCARSLIVD~TLPMHKQQ~DGAAWA~SRMSAIGHILGYGAGAIDLVA
NCU09321.1	FAIN-------~--------AD1TLPIQKQQ~SGAAWA1SRMGSLGHIIGYGMGAIDLLQ
FG01043.1 	FAINAGMIRFL1MSCSKSLIVD~TLPIEKQQ~SGAAWS1SRMSSIGHMIAYGAGAVDLIS
AN2982.1  	FAIN-------~----------~------VA1SSTSMC~SELNRR-YVTYTLATSWVRVG
          	****                            ..:: . *.:.   ::      :   : 

MG04371.1 	~LLGTTLGETQFKQLTLIAASGILFFSTLTCWAVSERVLVAPA~PEPKGLVAGGERFKVP
NCU09321.1	~LFGTSLGDTQFKQLTVIAALGMLVTSSVTCWAVTERVLVTVR~PDPRRQSG---RFKVV
FG01043.1 	~IFGKTLGDTQFKQLTVISTVALLSSTALTCWAVTERVLLTSK~P--AKHEG---RFKVF
AN2982.1  	2IFGTALGDTQFKQMTVIAALALLGAIAVTSYSVKERILITAR2D-SDGKAG---AFQVV
          	 ::*.:**:*****:*:*:: .:*   ::*.::*.**:*::     .    .    *:* 

MG04371.1 	RQIWSTLMTLPVRIQAICWAVFWSWIG~WYPFLIY0SSTWVGETYFRYDVPADAKSSSDA
NCU09321.1	RQIVSTLITLPPRIRAICYAVFWSWIG~WFPFIIY~SSTWVGETYFRYDVPADTRDSSDA
FG01043.1 	RQIWSTLLNLPPRIQAICWAQFWAWIG~WFPFLFY~STTWVGETYFRYDVPADARKSEDT
AN2982.1  	TQLFRTTMDLPPRIQAICWAQFWAWIG1WFPFLFY~STTWVGETYFRYEVPKDADRPVDM
          	 *:  * : ** **:***:* **:*** *:**::* *:**********:** *:  . * 

MG04371.1 	LGEMGRIGSYALTVYSIITFLGAWLIPPLVRSPEDDDEDDDGIPVTSQAAKSQSFLHSWF
NCU09321.1	LGDMGRIGSTALTVYSTVTFISAWILPPFIRAPEDN-----------------TFTHRPP
FG01043.1 	LGAIGRIGSTALVMYSVITFAGAWVLPMLVQSPEDN-----------------TFTHRPP
AN2982.1  	LGEVGRVGSLSLVVFSSITFISSVLLPFCVQSPDTKR---------------ARFTPRPP
          	** :**:** :*.::* :** .: ::*  :::*: .                  *     

MG04371.1 	PGVARSLGQLGHLKPDLLTVWTFGYIMFAFAMFMAPLASSFRFATILVALCGI~PWTISM
NCU09321.1	ASIASLVETVNKYKPELLSAWIASHLMFAFAMFLTPFAASFRFATALVALCGI~PWSIAI
FG01043.1 	QAIAGLLTSFNKLKPDLLTAWVFGHLMFAASMALAPFATSFRFATVLVCLCGI2PWTLAM
AN2982.1  	PGIAALLTKFMKIRPDLQTTWLISHVVFAATMIFAPLARSRAFATFLVAICGI~PWAVSC
          	 .:*  :  . : :*:* :.*  .:::** :* ::*:* *  *** **.:*** **::: 

MG04371.1 	WAPTALMGVEVNRMTHGITGQPYRRLSTSSAVELLTPVIRVNGEETEVVAAAAAPSS--A
NCU09321.1	WAPTTFLGVEVNKLSG--APDPMS-PGATSATNPSYRRLSTSSIEMRFVNEPAAG-----
FG01043.1 	WAPSAFLGVEVNKMSG-----------ETGADGTYRRISDEPDIELSEVSHNDAPLH--L
AN2982.1  	WAPFAFMGVEINRLAMN-PSQASRLSGVTMITSSSIRSGAYGDLNSSDASEMDVLRLNHH
          	*** :::***:*::: . . ..    . :             . :   .    .   .  

MG04371.1 	PQAALLEDGNGTRVGGGGGELSGIYFGILNIYTTLPQFIGTFISAVVFAILEPGKSPELS
NCU09321.1	-AVEEVTAGNSS-----TGELSGIYFGILNIYTTIPQFIATFMSSIVFAILEPGKSPELA
FG01043.1 	DHGSEVDLPSTAS----TGELSGIYFGILNIYTTLPQFVGTFISTIVFAILEPGKSPELA
AN2982.1  	DQDDDSDAEDGISNIPSTGELAGIYLGVLNVYTTLPQFVGTFISWIVFSILEPG-STKRD
          	         .      . ***:***:*:**:***:***:.**:* :**:***** *.:  

MG04371.1 	HGGGGEELPQAPSDGPNAIGICLFIGAISAVGAAYATRRLRYL-
NCU09321.1	TDAHPSEH--AKTDGPNAIAVCMFIGAISSLVAAYATAKMRDI-
FG01043.1 	DDAKKQPD----PDGPNAIAVCLFIGAMSSVVAAYVTRKLKVM-
AN2982.1  	DSASDSTWMDLDKSTPNAISICLFIGALCALIAMEATRRMRYIL
          	 ..  .   .   . ****.:*:****:.:: *  .* ::: :
```
